# The UV Photodissociation Spectrum of FeOH^+^: Electronic Insight into the Simplest Iron Hydroxide Complexes

**DOI:** 10.1021/acs.jpca.5c06546

**Published:** 2025-11-07

**Authors:** Shan Jin, Marcos Juanes, Marc Reimann, Christian van der Linde, Milan Ončák, Martin K. Beyer

**Affiliations:** † Universität Innsbruck, 27255 Institut für Ionenphysik und Angewandte Physik, Technikerstraße 25, Innsbruck 6020, Austria; ‡ Departamento Química Física y Química Inorgánica, 201461 University of Valladolid, Paseo de Belén 7, Valladolid 47011, Spain

## Abstract

FeOH^+^ has been proposed to exist in cold interstellar environments due to the high cosmic abundance of hydrogen, oxygen, and iron. In this study, we report the UV photodissociation spectrum of FeOH^+^ in the photon energy range 2.5–5.9 eV, complemented by high-level quantum chemical calculations. Previous studies focused on determining the bond dissociation energy of FeOH^+^ through measuring the threshold energy for its photodissociation into Fe^+^ and OH. Our observed photodissociation threshold of 3.47 ± 0.02 eV is an upper limit for the Fe^+^–OH bond dissociation energy and agrees within error limits with recent collision-induced dissociation data. In addition, the spectra provide insight into electronically excited states and quantitative photodissociation cross sections. The experimental band positions agree very well with theoretical calculations on the EOM-CCSD/aug-cc-pVTZ level in the energy range of 3.45–4.25 eV, while the measured photodissociation cross sections <3 × 10^–19^ cm^2^ are an order of magnitude smaller than the calculated absorption cross sections. The overall broad electronic transitions of FeOH^+^ in this region exhibit a weak substructure with an energy spacing in the range of calculated frequencies of the Fe–O stretching mode in various electronic states, which hints at vibrational progressions. After a gap of about 0.75 eV, both experiment and theory exhibit a second broad absorption feature above 5.0 eV. At these high energies, experimental photodissociation and calculated absorption cross sections show better agreement. The measured photodissociation cross sections allow estimation of the lifetime of FeOH^+^ in interstellar environments with respect to photodissociation, which is key data for the integration of the molecule in models of interstellar chemistry.

## Graphical abstract

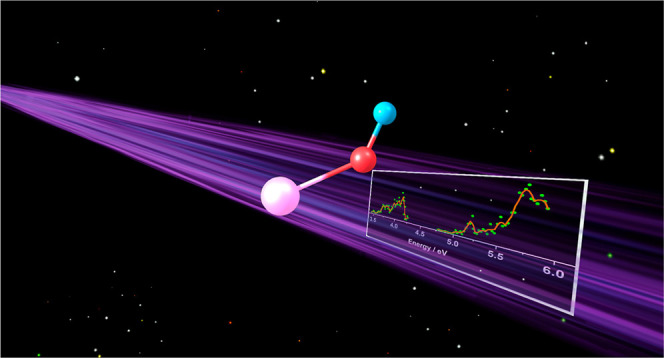

## Introduction

The hydroxyl radical OH, as the first molecule detected by radio astronomy in the interstellar medium (ISM),^1,2^ is widely found in the galactic ISM and local molecular clouds.^3–5^ As a highly reactive radical, OH also plays a crucial role in astrochemical reactions.^6,7^ In association with other atoms or molecules, species such as HOC^+^,^8^ HOCO^+^,^9^ HOCN,^10^ CH_3_OH,^11^ NH_2_OH,^12^ and the metal compound AlOH^13^ are formed. Interestingly, OH radicals react with condensed carbon monoxide (CO) on grain surfaces to HOCO, which either decomposes to carbon dioxide (CO_2_) and atomic hydrogen or reacts with atomic hydrogen to yield CO_2_ + H_2_.^14^ Dust grains are also widely seen as a sink for the majority of iron in the ISM.^15^

Due to its high cosmic abundance, iron has caught attention in astrochemistry.^16–20^ However, the detection of gas-phase iron-bearing molecules is limited to a tentative identification of FeO in Sgr B2 ^16^ and FeCN in IRC 10216.^17^ For decades, studies motivated by the search for iron-containing complexes in the ISM have been conducted intermittently, e.g., Fe-nanoparticles,^18^ Fe-pseudocarbynes,^21^ Fe-polycyclic aromatic hydrocarbon (PAH),^22–24^ FeCO,^25^ FeCO^+^,^26^ Fe^+^(H_2_O),^27^ Fe^+^(H_2_/D_2_),^28^ and FeH^+^.^29–35^ Duncan and co-workers measured photodissociation spectra of Fe^+^(C_2_H_2_) and Fe^+^(C_6_H_6_)_1,2_ complexes^36,37^ and extracted photodissociation thresholds to determine the bond dissociation energy. We conducted the fundamental and overtone infrared (IR) spectroscopy of the Fe–H stretch in Ar_2_FeH^+^, including high-level calculation for benchmarking.^38,39^ Compared to observations of Cygnus X-1, the laboratory X-ray absorption spectrum of the L_2,3_ edges of FeH^+^ provided neutral evidence, neither confirming nor refuting its presence in the ISM.^29^

FeOH^+^, the simplest iron hydroxide cation, was recommended to be a candidate for detection in the ISM.^27,40^ Experimentally, the bond dissociation energy D_0_(Fe^+^–OH) of FeOH^+^ was determined consistently in previous research. Murad measured D_0_(Fe^+^–OH) = 3.3 ± 0.2 eV using high-temperature mass spectrometry and ionization energy measurements.^41^ Cassady and Freiser obtained D_0_(Fe^+^–OH) = 3.34 ± 0.26 eV by the relative proton affinity of FeO, in agreement with their measured photodissociation threshold of D_0_(Fe^+^–OH) = 3.17 ± 0.13 eV.^42^ Sander and Armentrout’s collision-induced dissociation (CID) experiments yield D_0_(Fe^+^–OH) = 3.34 ± 0.18 eV.^43^ In contrast, Magnera, David, and Michl proposed the dissociation of Fe^+^–OH to the Fe^+^(^4^F) excited-state asymptote.^44^ Assuming the lowest-lying spin–orbit coupled states for quartet and sextet Fe^+^, respectively, their result corresponds to D_0_(Fe^+^(^4^F_9/2_)–OH) = 3.70 ± 0.13 eV and a ground-state bond dissociation energy of D_0_(Fe^+^(^6^D_9/2_)–OH) = 3.47 ± 0.13 eV. Schröder and Schwarz suggested possible reaction pathways (1) and (2) to form FeOH^+^.^45^ Although both reaction pathways are endothermic and require rare and special conditions, they also occur under some harsh conditions, e.g., chemical ionization plasma.

1 $${Fe}^{+}+H_{2}O\to {FeOH}^{+}+H$$

2 $${FeO}^{+}+H_{2}O\to {FeOH}^{+}+OH$$

Meanwhile, Gerlich, Roithová, and co-workers^40^ observed reaction (3) at cryogenic conditions, with low yield. Given the high cosmic abundance of the OH radical, we suggest that radiative association (4) might be a viable pathway to FeOH^+^ formation.

3 $${FeO}^{+}+H_{2}\to {FeOH}^{+}+H$$

4 $${Fe}^{+}+OH\to {FeOH}^{+}+h\nu$$

So far, spectroscopic research on FeOH^+^ is limited. Gutsev and Bauschlicher explored its geometric structure and calculated vibrational frequencies on the BPW91/6-311+G** level. They found that the energy of OH dissociatively attached to Fe^+^ (HFeO^+^) is significantly above the energy of OH associatively attached to Fe^+^ (FeOH^+^) by 2.23 eV.^46^ Cassady and Freiser conducted a photodissociation study on FeOH^+^ with two absorption maxima in the UV region, arguing that the bands are caused by the metal ion rather than by the metal–ligand charge transfer.^42^ Gerlich et al. demonstrated the first infrared spectra of He–FeOH^+^ in the O–H stretching region using an innovative wire quadrupole cryogenic trap. The spectra exhibited a double peak at 3694.5 cm^–1^ and 3697.4 cm^–1^, closely matching the anharmonic frequency of 3693 cm^–1^ calculated for the O–H stretch of He.FeOH^+^.^40^ However, the origin of the double peak could not be resolved.

Here, we use a high-resolution Fourier-transform ion cyclotron resonance (FT-ICR) mass spectrometer coupled with a tunable UV–vis laser in the spectral range of 2.5–5.9 eV to measure the photodissociation spectrum of the gas-phase molecular ion FeOH^+^. Quantum chemical calculations are performed to simulate the electronic transitions. Broad bands are observed together with a narrower feature on the lower energy side, which matches the upper limit of the dissociation threshold of the title complex. Absolute photodissociation cross sections are provided, which allow for the calculation of the photodissociation lifetime in interstellar environments, provided the photon flux in that area is known. This is critical data for the inclusion of FeOH^+^ into astrochemical reaction networks.

### Experimental and Computational Methods

Photodissociation spectroscopy was conducted on a modified 4.7 T Bruker Spectrospin CMS47X FT-ICR mass spectrometer,^47,48^ coupled with an EKSPLA NT 342B tunable optical parametric oscillator (OPO), described in detail before.^49–51^ Cationic iron hydroxide was produced in a laser vaporization source^52–54^ equipped with a Quantum Light Q2D33-1053 Nd:YLF laser (526.5 nm, max 25 mJ per pulse, 33.3 Hz) focusing on a rotating isotopically enriched iron disk (^56^Fe, U.S. DOE) and supersonic expansion of the plasma in helium seeded with water and N_2_O. The ions were guided through electrostatic optics to the ICR cell, where the temperature of the environment is controlled at ca. 80 K to minimize the contribution of blackbody infrared radiative heating.^38,55–59^

Photodissociation spectra were recorded by scanning the tunable laser system from 210 to 500 nm. The tunable laser has a pulse repetition rate of 20 Hz, with a bandwidth of ∼5 to 8 cm^–1^ as specified by the manufacturer. The laser wavelength is calibrated using an Ocean Optics Flame miniature spectrometer, which recorded the OPO signal wavelength during the experiment. The photodissociation cross section was derived from the experimental data as described in detail before.^60^

Quantum chemical calculations of the complexes were performed to complement the experiment. Structures and relative energies of complexes were optimized and calculated using density functional theory (DFT) and coupled cluster methods at the B3LYP/aug-cc-pVTZ and CCSD/aug-cc-pVTZ levels of theory, respectively. Bond dissociation energies (BDEs) were determined at the CCSD/aug-cc-pVTZ level of theory. Excitation energies were calculated using time-dependent density functional theory (TDDFT) and the equation of motion coupled-cluster (EOM-CC) methods based on B3LYP/aug-cc-pVTZ and CCSD/aug-cc-pVTZ in Gaussian.^61^ Energies of electronic excited states are further examined by multireference configuration interaction including the Davidson correction (MRCI+Q) with the same aug-cc-pVTZ basis set in Molpro 2023.2.^62^ The geometric parameters used here were extracted from the CCSD/aug-cc-pVTZ level of theory. Multireference calculations were performed based on state-averaged CASSCF wave functions using 21 roots of A′ and 20 roots of A″ symmetry, respectively. The active space included the 3d and 4s orbitals of Fe^+^ as well as the 2p orbitals of O, leading to a CAS (12,9). The resulting natural orbitals are depicted in Figure S1. All CCSD, EOM-CCSD, and MRCI calculations employed the frozen core approximation with the default core of the 1s orbital for oxygen and 1s through 3p orbitals for iron (10 orbitals in total).

## Results and Discussion

### Experimental Spectra

Photodissociation spectra of FeOH^+^ are shown in Figure 1 as well as in Figures S2–S4. FeOH^+^ fragments to Fe^+^ via loss of OH, which is consistent with Cassady and Freiser’s work.^42^ A three-point running average is presented as red curves. In the energy range 3.45–4.25 eV (Figure 1a), the absorption bands are observed with a cross section below 3 × 10^–19^ cm^2^. A very weak peak is detected at ca. 3.5 eV, with fragments emerging at 3.48 ± 0.02 eV (Figure S5) from the detection limit. This photodissociation threshold is an upper limit of the Fe^+^–OH bond dissociation energy. It is consistent with our calculated dissociation energy of 3.38 eV (see Table S1) and agrees well with the most recent experimental value of Sander and Armentrout, 3.34 ± 0.18 eV.^43^ Following the weak absorption, two well-separated bands are observed, each consisting of multiple substructures, within the energy regions of 3.6–3.9 eV and 3.9–4.2 eV. The substructures display nearly uniform energy spacings of approximately 0.1 eV (800 cm^–1^), which are indicative of vibrational progressions of the Fe–O stretching mode. In Figure 1b, the spectral patterns are observed with cross sections of 1–5 × 10^–18^ cm^2^. The absorption maximum peaks at 5.70 eV. A weaker band is recorded with a maximum at 5.20 eV.

**Figure 1:**
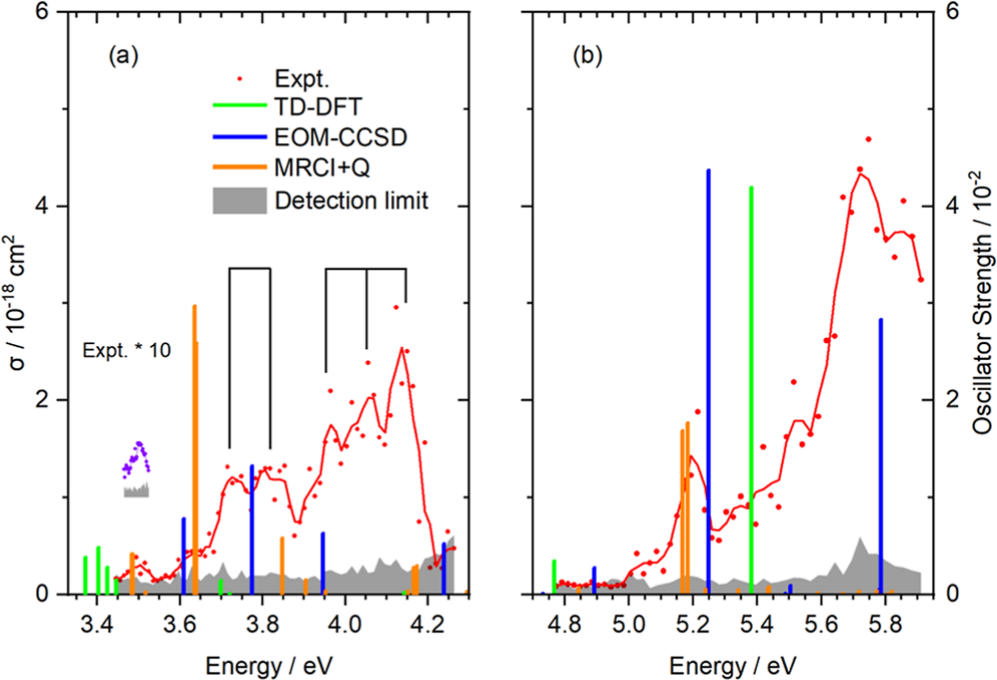
Photodissociation spectrum of FeOH^+^ in the UV range, together with three-point running average (red curves), calculations obtained from TDDFT methods at the B3LYP/aug-cc-pVTZ level, EOM-CCSD/aug-cc-pVTZ level, and MRCI+Q (12,9)/aug-cc-pVTZ level. The experimental spectrum in (a) is scaled up by a factor of 10 for better visibility. A precise scan (purple) is performed in the energy region of 3.45–3.52 eV, shown with an offset of 1 × 10^–19^ cm^2^. Groups of black vertical lines denote vibrational progressions, which are tentatively assigned to the Fe–O stretch.

In Figure S6, we extracted the data points from Cassady and Freiser’s work^42^ in comparison with our experimental spectra. Our spectrum partly agrees with the previous study in the overall energy range but provides significantly more spectral details and possible vibrational progressions that were not resolved before. In addition, our photodissociation threshold is significantly shifted to the blue. Cassady and Freiser noted that FeOH^+^ readily photodissociates, which is at odds with the low photodissociation cross section of <3 × 10^–19^ cm^2^ at photon energies below 4.5 eV. Since we repeated the spectrum several times, this work was conducted at a cooled ICR cell at ca. 80 K, and the laser system used by us delivers tunable light (<5 cm^–1^ resolution) in a more controlled way than the lamp and monochromator setup (10 nm resolution, about 600–1000 cm^–1^) used in the pioneering work by Cassady and Freiser,^42^ we are quite confident that our results are correct. The difference may in part be due to ion generation. Cassady and Freiser used laser ablation without supersonic expansion, which may lead to inefficient quenching of excited spin–orbit states of Fe^+^ and its reaction products with nitromethane.

### Theoretical Calculations

Table 1 presents the ground-state geometry of FeOH^+^ in the quintet state as well as the geometries of the lowest-lying triplet and septet states and the linear geometry of the quintet state, which features imaginary frequencies for the degenerate bending mode. The calculated energies for triplet and septet states lie 1.25 and 1.99 eV above the energy of the quintet state at the CCSD/aug-cc-pVTZ level. The energy for the linear structure of FeOH^+^ lies only 0.01 eV (∼80 cm^–1^) above the minimum, which is significantly less than the predicted OH bending frequency (395 cm^–1^) and thus significantly below the zero-point energy of the vibration, calculated at the CCSD/aug-cc-pVTZ level, pointing toward a floppy character of the molecule with respect to the Fe–O–H angle.

**Table 1:** Geometric Parameters and Relative Energies of FeOH^+^ Calculated Using B3LYP and CCSD Methods with the aug-cc-pVTZ Basis Set, Respectively

B3LYP	FeOH^+^ (^3^A′)	FeOH^+^ (^5^A′)	FeOH^+^ (^7^A′)	linear FeOH^+^ (^5^Δ)^a^
relative energy/eV	1.22	0	2.20	0.01
Fe–O bond length/Å	1.72	1.72	1.73	1.70
O–H bond length/Å	0.97	0.97	0.97	0.96
Fe–O–H angle/°	133	139	175	180

CCSD	FeOH^+^ (^3^A′)	FeOH^+^ (^5^A′)	FeOH^+^ (^7^Σ^+^)	linear FeOH^+^ (^5^Δ)^a^
relative energy/eV	1.25	0	1.99	0.01
Fe–O bond length/Å	1.73	1.73	1.70	1.70
O–H bond length/Å	0.96	0.96	0.97	0.95
Fe–O–H angle/°	137	139	180	180

a Second-order saddle point.

Calculation of the excitation energies of FeOH^+^ was performed using time-dependent DFT (B3LYP/aug-cc-pVTZ), equation-of-motion CCSD (aug-cc-pVTZ basis), and MRCI+Q/aug-cc-pVTZ, using a large active space of 12 electrons in 9 orbitals. The Davidson correction (+Q) is included to restore approximate size consistency; see computational details. The results are presented in Tables 2 and S2–S6. Extensive calculations on neutral FeOH by Hirano and Jensen^63,64^ have shown that the ground electronic state of linear FeOH is degenerate, resulting in a bent geometry and splitting of the degenerate ground-state levels due to the Renner–Teller effect.^65^ In the cationic form FeOH^+^, a similar behavior is observed. The MRCI calculation shows that two low-lying states, X^5^A′ and A^5^A″, are separated by an energy difference of merely 48 cm^–1^ (Table S5) in the ground-state minimum. Although our calculations are performed within the Born–Oppenheimer approximation, the observed small splitting hints at a degenerate ground state that would show a Renner–Teller splitting in a vibronic treatment. As the correct treatment of this effect is well outside the scope of this work, we simply refer to FeOH^+^ as a floppy, quasi-linear molecule. In our opinion, this results in an overall spectral broadening, as discussed below.

**Table 2:** Excitation Energies Δ*E* and Oscillator Strength of Bright Quintet-State Excitations Relative to the Ground State of FeOH^+^ ^a^

excited state	Irrep	Δ*E*(TDDFT)/eV	oscillator strength
6	A″	3.18	0.0031
7	A″	3.37	0.0038
8	A′	3.40	0.0048
9	A′	3.42	0.0028
16	A′	4.62	0.0231
20	A′	5.38	0.0419

excited state	Irrep	Δ*E*(EOM-CCSD)/eV	oscillator strength
6	A″	3.61	0.0078
7	A′	3.77	0.0132
8	A″	3.95	0.0063
9	A′	4.24	0.0052
14	A′	5.25	0.0437
18	A″	5.79	0.0283

excited state	Irrep	Δ*E*(MRCI+Q)/eV	oscillator strength
5	A′	3.48	0.00418
6	A″	3.49	0.00415
8	A′	3.64	0.02969
9	A″	3.64	0.02586
10	A″	3.85	0.00581
11	A′	3.90	0.00150
21	A′	4.66	0.00160
23	A′	5.17	0.01684
24	A″	5.18	0.01762
25	A′	5.24	0.00052
26	A′	5.34	0.00046
27	A″	5.43	0.00067

a TD-B3LYP/aug-cc-pVTZ results are based on a B3LYP/aug-cc-pVTZ-optimized geometry, whereas EOM-CCSD/aug-cc-pVTZ and MRCI+Q/aug-cc-pVTZ results are based on a CCSD/aug-cc-pVTZ-optimized geometry. States are numbered with increasing energy, i.e., excited state 1 denotes the first excited state.

## Discussion

Our experimental spectra can be interpreted by theoretical calculations pretty well. The excitation energies calculated using TDDFT, EOM-CCSD, and MRCI+Q methods are shown in Figure 1 as green, blue, and orange bars, respectively. Vertical excitation energies are listed in Tables 2 and S3–S5. In general, the calculated excitation energies of EOM-CCSD and MRCI+Q at the low energy side align well with the experimental bands at 3.6–3.9 and 3.9–4.2 eV. In particular, the MRCI+Q method predicts that a lower excitation energy at ∼3.48 eV is consistent with the experimental onset. The following excitation energies capture the experimental band positions in the higher energy range. EOM-CCSD produces strong excitations near 5.25 and 5.8 eV, consistent with the main experimental features. MRCI+Q yields two intense excitations near 5.18 eV, in reasonable agreement with the low-energy shoulder of the experimental band.

We note explicitly that all electronic states of FeOH^+^ show a pronounced multireference character (see Table S8 for the dominant contributions of the CAS-CI vector). The excellent agreement between EOM-CCSD and the experimental results is therefore at least partly due to error compensation. The same holds true for the TD-DFT calculations, which also predict excitation energies and oscillator strengths in the experimentally observed range, but the agreement is less pronounced.

Vibrational frequencies of bright electronically excited states of FeOH^+^ are calculated using TDDFT at the B3LYP/aug-cc-pVTZ level, see Table 3. The Fe–O stretching mode of FeOH^+^ exhibits frequencies of 791 cm^–1^ and 688 cm^–1^ in the sixth and seventh excited states, respectively, closely matching the progressions observed in Figure 1a. This strengthens the assignment of the spectral substructure to vibrational progressions in bound electronically excited states, most likely caused by the Fe–O stretching mode. Since we observe photofragmentation, excitation into bound states implies predissociation. In other words, internal conversions or intersystem crossings are required to reach the ground-state dissociative asymptote. Above 5 eV, the spectrum is overall broader, lacking vibrational progressions, which is more consistent with excitation into one or more repulsive states. This might also explain why the experimental photodissociation cross section is an order of magnitude lower below 4.2 eV, while the experimentally predicted absorption cross section is comparable. In the low energy range, predissociation competes with fluorescence after excitation to a bound state. The overall increase of the photodissociation cross section with photon energy indicates that the probability for predissociation increases with excitation energy. In this scenario, most excited FeOH^+^ ions undergo fluorescence at the observed photodissociation threshold, and the branching ratio gradually shifts toward predissociation with increasing excitation energy.

**Table 3:** Unscaled Vibrational Frequencies of FeOH^+^ in the Electronic Ground and Selected Excited Quintet States Using DFT and TDDFT at the B3LYP/aug-cc-pVTZ Level

	ν/cm^–1^				
	ground state/A′	6/A″	7/A″	8/A′	9/A′
Fe–O–H ben./cm^–1^	414	390	415	341	399
Fe–O str./cm^–1^	801	791	688	848	1054
O–H str./cm^–1^	3793	3735	3659	3662	3775
vertical excitation energy/eV	0	3.18	3.37	3.40	3.42
adiabatic excitation energy/eV	0	2.09	3.23	3.21	2.26

Figure 2 compares the calculated spectra of the bent and linear geometries of FeOH^+^. The details of excitation energies are presented using EOM-CCSD and MRCI+Q with the aug-cc-pVTZ basis set in Tables S6 and S7 of Supporting Information. Transitions are found in a similar range but with significant shifts, which suggests that the floppy nature of the FeOH^+^ geometry with respect to the bending mode causes significant spectral broadening. This would explain why the vibrational structure of the bands is only partly resolved.

**Figure 2:**
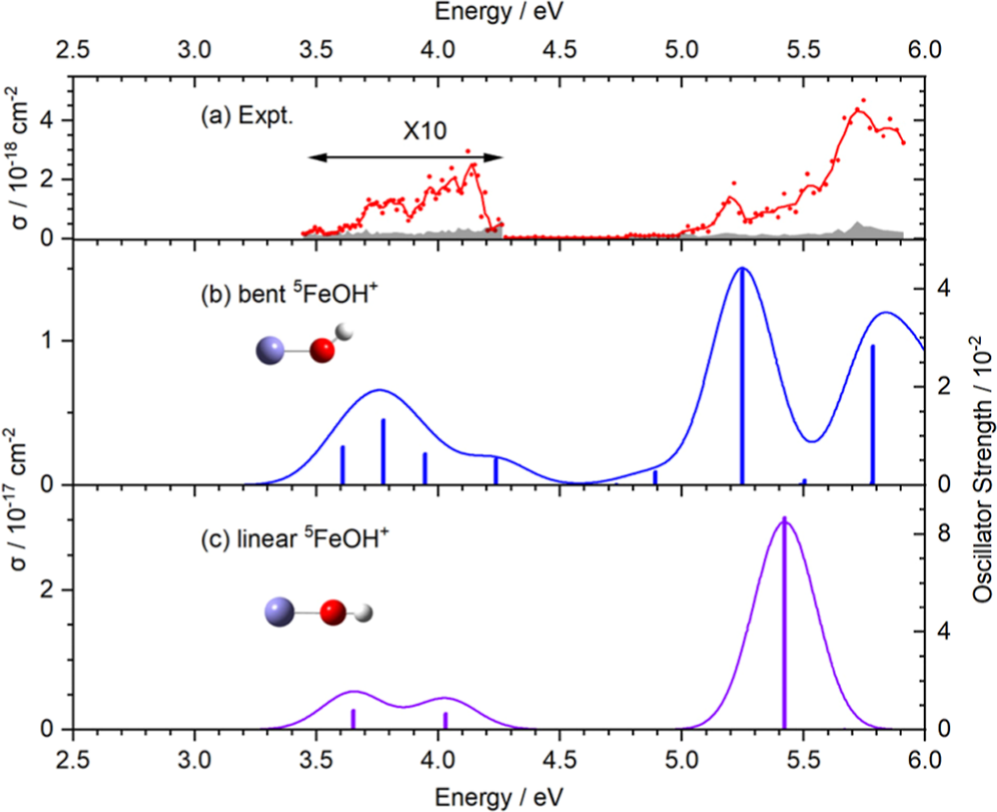
A comparison between the (a) experimental spectrum and (b,c) EOM-CCSD calculated spectra with the aug-cc-pVTZ basis set for FeOH^+^. Structure in (b) was optimized at the CCSD/aug-cc-pVTZ level for the ground state. The linear structure in (c) was optimized at the CCSD/aug-cc-pVTZ level and corresponds to a second-order saddle point.

## Conclusions

In this study, we have analyzed the electronic structure of FeOH^+^, a molecule of astrochemical interest, using UV photodissociation spectroscopy combined with high-level quantum chemical calculations employing TDDFT, EOM-CCSD, and MRCI+Q methods. The spectra are overall consistent with a previous study in terms of shape and energy range, but more substructure is resolved, likely corresponding to vibrational progressions. Within the energy range 3.45–4.25 eV, the experimental spectrum displays a distinct substructure, with a spacing of ∼0.1 eV, consistent with TDDFT frequency analysis of the Fe–O vibrational mode, suggesting that they originate from Fe–O vibrational progressions in electronically excited states. Furthermore, absorption features were observed in the high-energy region above 5.0 eV, which is in agreement with theoretical predictions. The broad photodissociation bands of FeOH^+^ in the UV region are probably not suitable for an astronomic identification of this molecular ion. However, the photodissociation cross sections allow for calculation of photodissociation lifetimes in interstellar radiation fields, which is required for the inclusion of FeOH^+^ in astrochemical models.

## Supplementary Material





## Notes

Numerical data of experimental FeOH^+^ photodissociation spectra (XLSX)
Additional experimental data sets; determination of the photodissociation threshold; comparison with the literature spectrum; synopsis of bond dissociation energies; and additional computational details including benchmarks and optimized geometries (PDF)
