# Supplementary material for: The UV Photodissociation Spectrum of FeOH+: Electronic Insight into the Simplest Iron Hydroxide Complexes
Source: J Phys Chem A. 2025 Nov 7;129(46):10730–6. doi: 10.1021/acs.jpca.5c06546 (PMC12641485; doi:10.1021/acs.jpca.5c06546)
Supplement: Supplementary file 2 [file jp5c06546_si_002.pdf]

Supporting information

UV Photodissociation Spectrum of $FeOH^+$: Electronic Insight into the Simplest Iron Hydroxide Complexes

Shan Jin,[a] Marcos Juanes,[a,b] Marc Reimann,[a] Christian van der Linde,[a] Milan Ončák[a,] and Martin K. Beyer[a,*]*

[a] *Universität Innsbruck, Institut für Ionenphysik und Angewandte Physik, Technikerstraße 25, 6020 Innsbruck, Austria*

[b] *Departamento Química Física y Química Inorgánica, University of Valladolid, Paseo de Belén 7, 47011 Valladolid, Spain*

Email: milan.oncak@uibk.ac.at; martin.beyer@uibk.ac.at

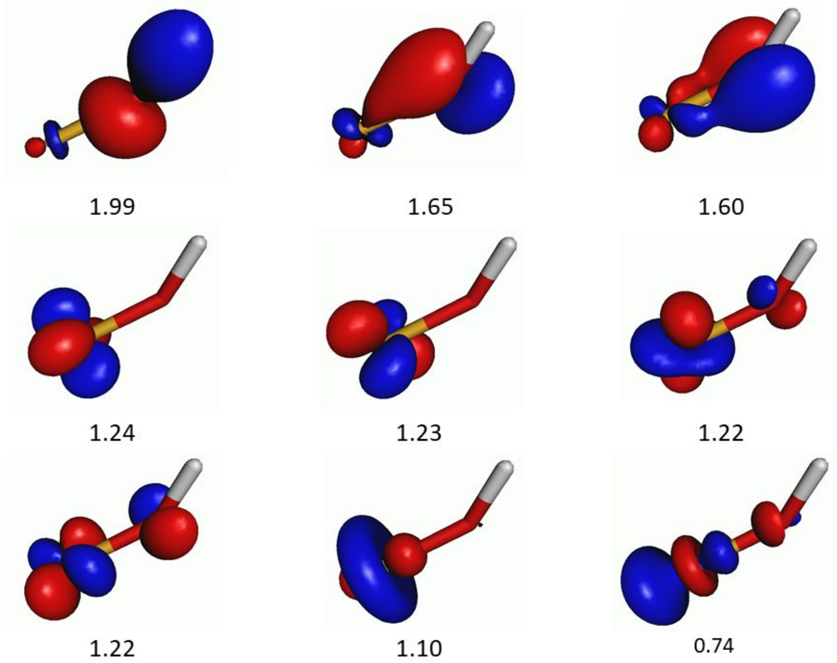

Figure S1. Natural orbitals and their occupation numbers from a SA-CASSCF(12,9) calculation as described in the main text. Isosurfaces are shown for a value of 0.01.

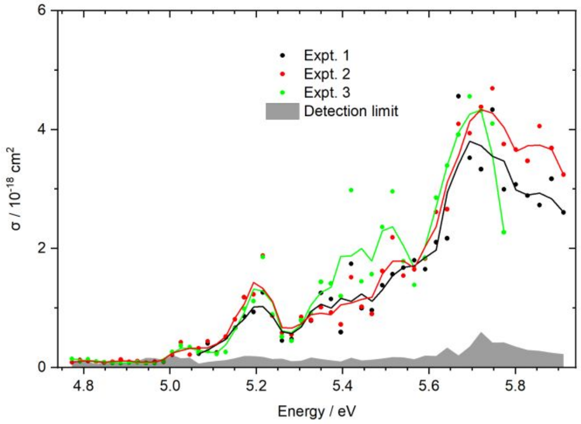

Figure S2. Reproducibility of the photodissociation spectra of $FeOH^+$ in the spectral region of 4.8–5.9 eV tested with three experimental runs.

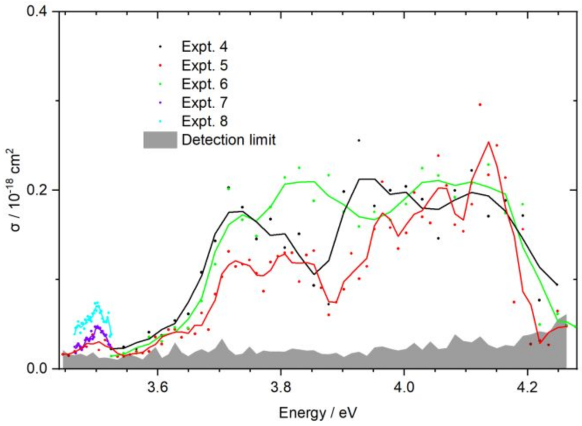

Figure S3. Reproducibility of the photodissociation spectra of $FeOH^+$ in the spectral region of 3.45–4.25 eV tested with experimental runs 4, 5 and 6. Exp. 7 and 8 are densely spaced scans of the onset of photodissociation.

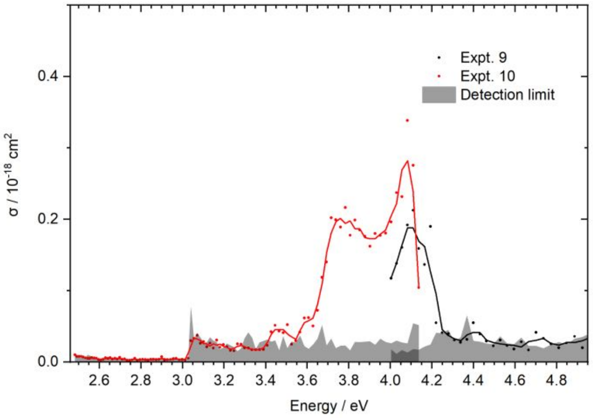

Figure S4. Two coarse scans of the $FeOH^+$ photodissociation spectrum in the spectral region of 2.5–5.0 eV.

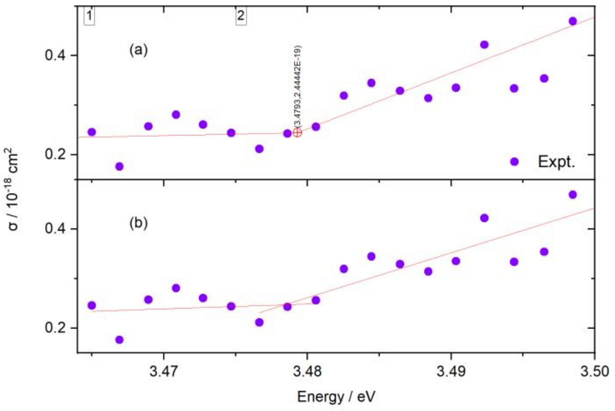

Figure S5. Photodissociation spectrum (Expt. 7 of Figure S2) of $FeOH^+$ in the respective threshold energy regions. Photodissociation threshold is determined using OriginPro (a) Onset of Slope and (b) two Linear Fits.

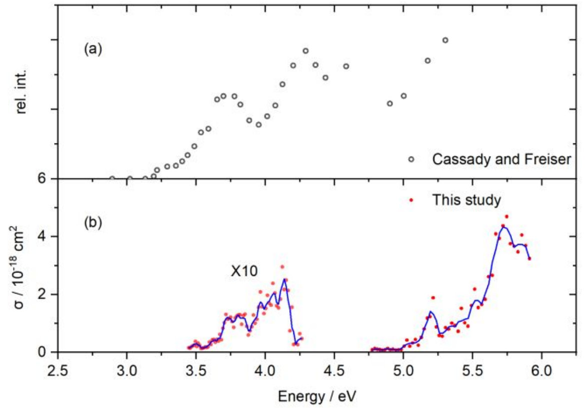

Figure S6. Comparison of $FeOH^+$ photodissociation spectrum measured by Cassady and Freiser[1] with this study.

Table S1. Bond dissociation energies (BDEs) for $FeOH^+$ ($^5A'$) calculated using CCSD method with aug-cc-pVTZ basis set, compared with the photodissociation threshold extracted from experimental spectra. Literature BDEs and photodissociation thresholds from previous studies are also included for reference.

Reactant		Products		[a]BDEs/ eV	BDE(Fe^+-OH)/ eV	Photodissociation threshold/ eV
$^5[FeOH]^+$	→	Fe^+ (6D)	OH	3.38	3.34 ± 0.26[1]<br>3.34 ± 0.18[2]<br>3.3 ± 0.2[3]	3.48 ± 0.02[a]<br>3.17 ± 0.13[1]
		Fe^+ (4F)	OH	3.65	3.70 ± 0.13[4]	-
		$^6[FeO]^+$	H	4.99	-	-
		$^4[FeO]^+$	H	5.85	-	-
		$^5[FeH]^+$	O	5.47	-	-

[a]This work

Table S2. Unscaled frequencies of $FeOH^+$ ($^5A'$) in the electronic ground state using CCSD/aug-cc-pVTZ level of theory.

vibrational mode	ν / cm^{-1}
O–H ben.	395
Fe–O str.	820
O–H str.	3914

Table S3. The excitation energies of $FeOH^+$ ($^5A'$) were calculated using the EOM-CCSD/aug-cc-pVTZ method.

Excited state	irrep	E / eV	E / cm^{-1}	f
1	A"	0.30	2423	0
2	A'	0.79	6347	0.0001
3	A"	1.03	8344	0.0002
4	A'	1.09	8794	0
5	A'	3.50	28221	0
6	A"	3.61	29112	0.0078
7	A'	3.77	30438	0.0132
8	A"	3.95	31832	0.0063
9	A'	4.24	34188	0.0052
10	A'	4.73	38166	0.0001
11	A"	4.74	38241	0
12	A'	4.89	39459	0.0027
13	A"	5.07	40903	0
14	A'	5.25	42337	0.0437
15	A"	5.49	44261	0.0001
16	A'	5.50	44397	0.0009
17	A"	5.78	46631	0.0005
18	A'	5.79	46664	0.0283
19	A'	6.01	48440	0.02
20	A"	6.75	54469	0

Table S4. The excitation energies of $FeOH^+$ ($^5A'$) were calculated using the TDDFT method based on B3LYP/aug-cc-pVTZ reference.

Excited state	irrep	E / eV	E / cm^{-1}	f
1	A"	0.25	1978	0
2	A'	0.74	5947	0.0001
3	A'	1.02	8237	0.0001
4	A"	1.02	8253	0.0001
5	A'	3.02	24379	0
6	A"	3.18	25668	0.0031
7	A"	3.37	27190	0.0038
8	A'	3.40	27452	0.0048
9	A'	3.42	27617	0.0028
10	A"	3.45	27796	0.0015
11	A'	3.70	29836	0.0014
12	A"	3.72	30004	0.0001
13	A'	4.14	33421	0.0002
14	A'	4.25	34295	0
15	A"	4.50	36311	0.0001
16	A'	4.62	37294	0.0231
17	A'	4.77	38445	0.0034
18	A"	4.77	38472	0.0001
19	A"	5.22	42072	0
20	A'	5.38	43410	0.0419

Table S5. The excitation energies of bent $FeOH^+$ ($^5A'$), along without and with Davidson correction calculated at the MRCI (12,9)/aug-cc-pVTZ level and MRCI+Q (12,9)/aug-cc-pVTZ level, respectively. Calculations were performed using the CCSD/aug-cc-pVTZ optimized structure. Ground state 0 is listed for reference.

Excited state	Symmetry	*E(MRCI)* /eV	*E / cm⁻¹*	*f*	Symmetry	*E(MRCI+Q)* /eV	*E* / cm⁻¹	*f*
0	A'	0.00	0	--	A'	0.00	0	--
1	A''	0.00	25	--	A''	0.01	48	--
2	A'	0.57	4578	0.00004	A'	0.53	4297	0.00003
3	A'	0.84	6746	0.00003	A''	0.82	6621	0.00009
4	A''	0.85	6884	0.00009	A'	0.87	7015	0.00003
5	A'	2.90	23407	0.00348	A'	3.48	28096	0.00418
6	A''	2.90	23419	0.00345	A''	3.49	28116	0.00415
7	A''	3.12	25175	0.02220	A'	3.52	28366	0.00021
8	A'	3.13	25271	0.02559	A'	3.64	29321	0.02969
9	A'	3.13	25273	0.00019	A''	3.64	29351	0.02586
10	A''	3.26	26321	0.00493	A''	3.85	31033	0.00581
11	A'	3.36	27128	0.00130	A'	3.90	31486	0.00151
12	A'	3.48	28048	0.00032	A'	3.95	31881	0.00037
13	A''	3.62	29234	0.00030	A''	4.15	33491	0.00034
14	A''	3.67	29595	0.00247	A''	4.17	33598	0.00280
15	A'	3.67	29628	0.00258	A'	4.17	33656	0.00293
16	A''	3.75	30234	0.00028	A''	4.29	34638	0.00032
17	A'	3.91	31546	0.00302	A'	4.40	35491	0.00340
18	A''	3.91	31575	0.00319	A''	4.41	35564	0.00359
19	A''	4.01	32320	0.00073	A'	4.47	36093	0.00006
20	A'	4.05	32649	0.00006	A''	4.51	36370	0.00083
21	A'	4.17	33668	0.00143	A'	4.66	37623	0.00160
22	A''	4.34	34983	0.00060	A''	4.84	39072	0.00067
23	A'	4.76	38362	0.01550	A'	5.17	41679	0.01684
24	A''	4.77	38457	0.01621	A''	5.18	41813	0.01762
25	A'	4.81	38803	0.00048	A'	5.24	42257	0.00052
26	A'	4.91	39626	0.00042	A'	5.34	43080	0.00046
27	A''	5.04	40646	0.00062	A''	5.43	43831	0.00067
28	A'	5.05	40703	0.00071	A'	5.44	43855	0.00077
29	A''	5.14	41476	0.00007	A''	5.59	45080	0.00013
30	A''	5.16	41632	0.00012	A''	5.67	45712	0.00008
31	A''	5.28	42561	0.00028	A''	5.72	46108	0.00030
32	A'	5.33	42967	0.00034	A'	5.77	46564	0.00037
33	A''	5.38	43426	0.00004	A''	5.81	46835	0.00004
34	A'	5.40	43556	0.00028	A'	5.82	46943	0.00030
35	A''	5.53	44615	0.00110	A'	6.00	48421	0.00055
36	A'	5.54	44646	0.00051	A''	6.01	48455	0.00119
37	A'	5.56	44883	0.00077	A'	6.04	48699	0.00083
38	A''	5.61	45235	0.00031	A''	6.10	49198	0.00034
39	A'	5.69	45915	0.00062	A'	6.21	50124	0.00068
40	A''	5.74	46280	0.00065	A''	6.25	50381	0.00071

Table S6. The excitation energies of linear $FeOH^+$ were calculated using the EOM-CCSD/aug-cc-pVTZ method. Degenerate states are counted as one state.

Excited state	irrep	E / eV	E / cm^{-1}	f
1	Σ^-	0.30	2413	0
2	Σ^+	0.96	7707	0
3	Π	1.04	8368	0.0002
4	Σ^+	3.37	27147	0
5	Π	3.65	29452	0.0079
6	Π	4.03	32515	0.0065
7	Σ^+	4.59	37019	0
8	Δ	4.89	39417	0
9	Σ^-	4.96	40010	0
10	Σ^+	5.42	43731	0.0867
11	Π	5.53	44641	0
12	Σ^+	5.67	45721	0
13	Σ^-	6.63	53433	0
14	Δ	7.54	60809	0

Table S7. The excitation energies of linear $FeOH^+$ ($^5\Delta$) calculated at the MRCI (12,9)/aug-cc-pVTZ level and MRCI+Q (12,9)/aug-cc-pVTZ level using Molpro. Calculations were performed using the CCSD/aug-cc-pVTZ optimized structure. Degenerate states are counted as one state.

Excited state	irrep	*E(MRCI)* /eV	*E / cm⁻¹*	*f*	irrep	*E(MRCI+Q)* /eV	*E* / cm⁻¹	*f*
0	Δ	0.00	0	0	Δ	0	0	0
1	Σ+	0.72	5806	0	Σ+	0.72	5844	0
2	Π	0.88	7116	0.00011	Π	0.81	6495	0.0001
3	Π	2.86	23087	0.00329	Σ+	3.32	26799	0
4	Π	2.87	23128	0.00341	Π	3.49	28155	0.00322
5	Σ+	2.95	23787	0	Π	3.5	28216	0.00333
6	Δ	3.22	26000	0.00028	Δ	3.87	31205	0.00028
7	Δ	3.37	27214	0	Δ	3.97	32025	0
8	Π	3.54	28580	0.00020	Π	4.15	33462	0.00019
9	Π	3.75	30261	0.00257	Π	4.31	34758	0.00252
10	Π	3.77	30407	0.00253	Π	4.33	34932	0.00248
11	Δ	4.17	33658	0.00135	Δ	4.75	38280	0.00136
12	Σ-	4.22	34051	0	Σ-	4.8	38746	0
13	Δ	4.58	36961	0.01973	Δ	5.05	40759	0.01979
14	Δ	5.01	40415	0	Π	5.51	44425	0
15	Π	5.02	40454	0	Δ	5.52	44513	0
16	Π	5.09	41052	0	Σ+	5.57	44946	0
17	Σ+	5.10	41142	0	Π	5.69	45860	0
18	Δ	5.35	43169	0	Δ	5.81	46826	0
19	Σ-	5.44	43843	0	Σ-	5.99	48315	0
20	Δ	5.49	44280	0.00024	Δ	6	48431	0.0002
21	Π	5.59	45096	0.00041	Π	6.18	49838	0.0004
22	Π	5.72	46117	0.00062	Π	6.3	50828	0.00061
23	Σ+	5.97	48130	0	Σ+	6.57	53021	0

Table S8 Dominant configuration interaction (CI) vectors and configuration interaction coefficients of $FeOH^+$ calculated at CASSCF (12, 9) /aug-cc-pVTZ level

CI vector	CI coefficients						
Symmetry A‘	1	2	3	4	5	6	7
22aa20 2aa		0.202246	0.782841				
222aa0 2aa	0.766235	0.318153					
22a2a0 2aa		0.688662					
22aaaa ba2					0.685838		
22aaaa 220				0.621505			
22aaaa 2ab					0.534492		
222a0a 2aa						0.394524	
2b2aaa 2aa	0.32159					0.455783	
2ba2aa 2aa		0.395495					
2baa2a 2aa			0.253129				0.413586
22baaa 2aa							0.20807
22a02a 2aa							0.243478
2baaa2 2aa							0.208598
	8	9	10	11	12	13	14
22aa20 a2a	0.719081						
2a2a20 2aa			0.63416				
22aa20 aa2				0.480674			
222a0a 2aa						0.361707	0.466884
2b2aaa 2aa		0.207736					
22a20a 2aa		0.365347					
22aaab a2a	0.416889						
22a2a0 a2a							0.224172
2ba2aa 2aa		0.413696		0.205239			
2baa2a 2aa					0.25509		
2aa220 2aa		0.271269			0.271915		
22aaab 2aa					0.287303	0.231411	
2a2aab 2aa			0.346774				
222aa0 aa2						0.284155	0.273668
2aaa2b 2aa					0.323196		
22aaab aa2				0.285018			
22abaa 2aa		0.246121					
22a02a 2aa					0.216663		
222a0a aa2							0.216855
2aa202 2aa				0.208657			
	15	16	17	18	19	20	21
22aaaa 220			0.251246				
2aaaa0 222							0.610826
22aaaa b2a			0.205126				

22a2a0 aa2	0.443355						
22a20a 2aa	0.429509						
22aaab a2a							
22a2a0 a2a			0.28657	0.415182			
22aaaa 2ba		0.411					
2aaa0a 222							0.386246
2a22a0 2aa		0.246947			0.381792	0.290523	
2220aa 2aa						0.334308	
22baaa 2aa				0.212747			
222aa0 aa2					0.332559		
22a20a aa2	0.331421						
2aaa2b 2aa					0.225879		
22a20a a2a				0.273822			
2220aa aa2						0.261141	
22aaaa a2b			0.257642				
22abaa aa2			0.256371				
22aaaa ab2		0.256111					
2a220a 2aa					0.249908		
22abaa 2aa			0.211364				
22aaba 2aa					0.217503		
222a0a aa2					0.215566		

Symmetry A‘‘	1	2	3	4	5	6	7
22aaa0 22a	0.827772				0.282766		
22aaa0 2a2		0.753701					
222a20 aaa							0.679493
22a2aa baa				0.596843			
222aaa 2a0			0.585604				
22a2aa 2a0				0.56819			
222aaa baa			0.564439	0.20294			
22aa2a baa						0.556328	
22aa2a 2a0						0.513084	
2baaaa 22a					0.475678		
222aab aaa							0.393947
22a0aa 22a					0.385651		
22aaa2 baa					0	0.23454	
22aaa2 2a0					0	0.23103	
	8	9	10	11	12	13	14
22aa2b aaa					0.789827		
22aaa0 2a2	0.32526						
2aaa20 2a2	0.375016			0.626242			
22aa0a 22a						0.587671	
2aaa20 22a			0.535226				

22a220 aaa		0.514287	0.431884				
2baaaa 2a2	0.40238						
2222a0 aaa							0.310256
222aaa 20a							0.424375
22aab2 aaa					0.399743		
22a0aa 22a							0.217406
2aaaab 2a2	0.20618			0.358088			
22a2ab aaa		0.335893	0.257933				
22a0aa 2a2	0.310726						
2aaaab 22a			0.300656				
22220a aaa							0.203533
	15	16	17	18	19	20	
222a20 aaa						0.219966	
222aaa 2a0			0.293562				
2222a0 aaa		0.453141					
2aa2a0 2a2	0.44001	0.416093					
2a2aa0 2a2					0.438599		
2aa2a0 22a				0.428571			
22aaa0 a22				0.313536			
22a0aa 22a			0.233399			0.378801	
22aa0a 2a2	0.297475	0.271998					
220aaa 22a					0.359027		
2aa20a 2a2	0.310118	0.28902					
2a2a0a 2a2					0.286531		
22220a aaa		0.282538					
2aa20a 22a				0.281333			
222baa aaa						0.238554	
222aba aaa			0.219323				
2a2aa0 22a	0.222412						

References

(1) Cassady, C. J.; Freiser, B. S. Determination of the Fe^{+}-OH and Co^{+}-OH bond energies by deprotonation reactions and by photodissociation. *J. Am. Chem. Soc.* **1984**, *106* (21), 6176–6179. DOI: 10.1021/ja00333a009.

(2) Sander, O.; Armentrout, P. B. Hydration Energies of Iron Hydroxide Cation: A Guided Ion Beam and Theoretical Investigation. *J. Phys. Chem. A* **2019**, *123* (8), 1675–1688. DOI: 10.1021/acs.jpca.8b12257. Published Online: Feb. 15, 2019.

(3) Murad, E. Thermochemical properties of gaseous FeO and FeOH. *J. Chem. Phys.* **1980**, *73* (3), 1381–1385. DOI: 10.1063/1.440255.

(4) Magnera, T. F.; David, D. E.; Michl, J. Gas-phase water and hydroxyl binding energies for monopositive first-row transition metal ions. *J. Am. Chem. Soc.* **1989**, *111* (11), 4100–4101. DOI: 10.1021/ja00193a051.

Cartesian coordinates (Å) of optimized structures along with their electronic energy (Hartree)

$^{3}FeOH^{+}$, B3LYP/aug-cc-pVTZ

E=-1339.289113

Fe	0.020151	-0.461367	0.000000
O	0.020151	1.259018	0.000000
H	-0.685137	1.923398	0.000000

$^{3}FeOH^{+}$, CCSD/aug-cc-pVTZ

E=-1338.079567

Fe	0.018727	-0.463874	0.000000
O	0.018727	1.262203	0.000000
H	-0.636722	1.963100	0.000000

$^{5}FeOH^{+}$, B3LYP/aug-cc-pVTZ

E= -1339.333879

Fe	0.018226	-0.462298	0.000000
O	0.018226	1.254919	0.000000
H	-0.619684	1.980401	0.000000

$^{5}FeOH^{+}$, CCSD/aug-cc-pVTZ

E=-1338.125708

Fe	0.017796	-0.464565	0.000000
O	0.017796	1.261101	0.000000
H	-0.605073	1.989872	0.000000

$^{7}FeOH^{+}$, B3LYP/aug-cc-pVTZ

E= -1339.252980

Fe	0.002438	-0.472565	0.000000
O	0.002438	1.257427	0.000000
H	-0.082880	2.227271	0.000000

$^{7}FeOH^{+}$, CCSD/aug-cc-pVTZ

E= -1338.052394

Fe	0.000000	0.000000	0.464441
O	0.000000	0.000000	-1.234431
H	0.000000	0.000000	-2.200010

Linear $^{5}FeOH^{+}$, B3LYP/aug-cc-pVTZ

E= -1339.333383

Fe	0.000000	0.000000	0.463862
O	0.000000	0.000000	-1.233092
H	0.000000	0.000000	-2.195680

Linear $^{5}FeOH^{+}$, CCSD/aug-cc-pVTZ

E=-1338.125284

Fe	0.000000	0.000000	0.465128
O	0.000000	0.000000	-1.237615
H	0.000000	0.000000	-2.192396
